# Antiviral Effect of Ginsenoside Rb2 and Rb3 Against Bovine Viral Diarrhea Virus and Classical Swine Fever Virus *in vitro*

**DOI:** 10.3389/fvets.2021.764909

**Published:** 2021-12-08

**Authors:** Bin Tan, Massimo Giangaspero, Na Sun, Yinping Jin, Kexin Liu, Qianying Wang, Shipeng Cheng, Yingping Wang, Shuqin Zhang

**Affiliations:** ^1^Institute of Special Economic Animal and Plant Sciences, Chinese Academy of Agricultural Sciences, Changchun, China; ^2^Faculty of Veterinary Medicine, University of Teramo, Teramo, Italy; ^3^National and Local Joint Engineering Research Center for Ginseng Breeding and Development, Changchun, China

**Keywords:** antivirus activity, ginsenoside Rb2/Rb3, BVDV, CSFV, *in vitro*

## Abstract

Bovine viral diarrhea virus (BVDV) and classical swine fever virus (CSFV) are members of the genus *Pestivirus* that cause disease in wild and domestic animals and are responsible for extensive economic losses of livestock and biological industry. BVDV is also a significant laboratory contaminant. Currently, no effective antiviral therapeutics are available to control their infection. Ginsenosides, as major pharmacological ingredients in the plants of ginseng, have various biological activities. In the present work, the antiviral activity of 9 ginsenosides and 3 other saponins from *Araliaceae* plants was investigated against *Pestivirus*. Ginsenoside Rb2 and Rb3 showed low cytotoxicity and obvious antiviral effect. They were able to inhibit the replication and proliferation of BVDV and CSFV. In addition, our results suggest that the possible antiviral mechanism of Rb2 might be related to its ability to affect the translation of these viruses. Obtained results suggest that ginsenoside Rb2 and Rb3 have a potential for effective treatment against *Pestivirus* infection.

## Introduction

Bovine viral diarrhea virus (BVDV) and classical swine fever virus (CSFV) are members of the genus *Pestivirus*, family *Flaviviridae*. They are viral pathogens affecting wild fauna and domestic livestock, causing extensive economic losses worldwide (1). Their genomes are single stranded positive polarity RNA, which consist of a single large open reading frame (ORF) flanked by 5′ and 3′ untranslated regions (UTR). The ORF encodes a polyprotein of ~3,900 aa and cleaved by viral and cellular proteinases into the individual viral proteins (2). The highly conserved 5′ UTR of genome binds to the host ribosome using the internal ribosome entry site (IRES) mechanism that facilitates translation of *Pestivirus* proteins (3).

CSFV infection mainly causes high fever, multiple hemorrhages, and leukopenia, leading to high morbidity and mortality, the severity of which might be due to the host species and the virulence of viral strains (4). Infections with BVDV are related to various clinical conditions such as reproductive failure, subclinical infection, persistent infections, severe acute disease with or without hemorrhagic diathesis, and even fatal mucosal disease (5, 6). BVDV is not only an important clinical pathogen, but also a significant laboratory contaminant. It has been reported that bovine serum, canine, bovine and feline cell lines, and vaccine commercially available have been found to be contaminated with BVDV (7–9).

Panax ginseng Meyer, a perennial herb of the *Araliaceae* family, has been used as a preventive and/or therapeutic herbal medicine in China, Japan, and Korea to strengthen holistic health for thousands of years (10). Ginsenosides are considered the major components of ginseng, they have a variety of biological activities, such as anti-aging, anti-oxidative, anti-cancer, and other actions improving the health (11). Currently, numerous studies have reported the beneficial effects on infections with pathogenic viruses. 20(R)-ginsenoside Rh2 was shown to suppress the replication of gamma herpesviruses in mouse and humans (12), 20(S)-protopanaxtriol possessed potent *in vitro* antiviral effect on the CVB3 (13). In particular, Re, Rf, and Rg2 could protect the host from the infections of rhinovirus 3 and coxsackievirus. In addition, ginsenoside Rg2 has also been found to have significant anti-EV71 activity (14). Ginsenoside Rg3 presented antiviral activity on Hepatitis C virus (HCV), inhibiting HCV-induced abnormal mitochondrial fission and mitophagy (supporting mechanisms for the establishment of persistent viral infection) (15). Rb2 showed the ability to reduce the virus titers and protect against infection of rotavirus and Sendai virus in mice (16, 17). However, to date, little is known about antiviral activity of the ginsenosides against BVDV. Currently, no effective antiviral therapeutics are available to control the BVDV infection. Thus, we have evaluated the antiviral activities of ginsenosides and other saponins for *in vitro* toxicity and activity against BVDV, also for a possible application to other closely genetically related pathogens as HCV.

## Materials and Methods

### Cells and Viruses

Madin Darby Bovine Kidney (MDBK) cells and swine testicle (ST) cells were grown in high-glucose Dulbecco's Modified Eagle Medium (DMEM), which was added with 8% fetal bovine serum (FBS). The FBS was confirmed to be free of BVDV antibody with commercial BVDV antibody test kit (IDEXX Laboratories, Inc.). All cells and FBS were tested negative for the presence of *Pestivirus* antigen by RT-PCR (18). The cpBVDV C24V and CSFV Shimen strains were obtained from the China Institute of Veterinary Drug Control, and propagated in MDBK and ST cells, respectively.

### Ginsenosides

Ginsenosides Rb1, Rb2, Rb3, Rc, Re, Rf, Rg1, Rh1, 20(S)-Rh2, pseudo ginsenoside Rh2 (PRh2), pseudo ginsenoside F11 (PF11), and NotoginsenosideR1 (NR1), screened in this study, were obtained from the Jilin University. The purity of these ginsenosides was above 98%, as estimated by high performance liquid chromatography. Stock solutions (40 mg/mL) of the compounds were dissolved in dimethyl sulfoxide (DMSO) or ethanol and were subsequently diluted in culture medium. As a negative control, DMSO was also added to all no-drug control samples.

### Cytotoxicity Assays

Cytotoxicity assays were carried out as described previously (19). In short, MDBK cells, exponentially grown in the 96-well plates (approximately 5 × 10^3^ cells/well), were incubated in 5% CO_2_ at 37°C for 24 h. Then, the medium was removed and serial dilutions of compounds were added. DMSO was used as a negative control. The cells were proliferated at 37°C for 3 days, and their overall metabolic activity was determined by the method of MTS/PMS (Promega, USA). The 50% cytotoxic concentration (CC_50_) was calculated using a non-linear regression fitting of the data as the compound concentration necessary to reduce 50% cell viability compared to control non-treated cells.

### Antiviral Screening Using CPE Inhibition Assay

The activity of compounds against BVDV was achieved by inhibiting the cpBVDV C24V strain induced cytopathogenicity in MDBK cells. Cells were seeded in 96-well plates and incubated overnight in growth medium in 5% CO_2_ at 37°C. Then, the cells were infected with virus at 10^3^ TCID_50_/mL, using a multiplicity of infection (MOI) of 0.05. After 2 h, the virus was removed and maintenance medium (DMEM supplemented with 2% FBS) with or without serial dilutions of compounds was added. Concentrations of compounds were from 6.25 to 200 μg/mL. DMSO was used as a control sample. The cells were incubated at 37°C for 3 days. Subsequently, the antiviral activity was evaluated through the viral titration and the cytopathic effect (CPE) inhibition, by MTS/PMS (Promega, USA). The 50% effective concentration (EC_50_) was calculated using a non-linear regression fitting of the data as the compound concentration necessary to reduce 50% cytopathic effect on MDBK cells compared to DMSO treated control cells.

### Antiviral Activity of Ginsenoside Rb2 and Rb3 Against cpBVDV Strain

MDBK cells were seeded in 6-well plates and cultured overnight, then inoculated with cpBVDV strain C24V with 0.05 MOI and Rb2 or Rb3 (200 μg/mL) mixture. Uninfected cells, added with DMSO, were used as controls. The plates were incubated for 72 h at 37°C to be further submitted for BVDV RNA and E2 protein detection by real-time PCR and Western blot.

### Real-Time Quantitative Polymerase Chain Reaction

Total RNA was extracted, retrotranscribed, and quantified by the real-time PCR. Following manufacturer's instructions, TRIzol reagent was used to extract RNA (Invitrogen, China) and the M-MLV Reverse Transcriptase was used to conduct the reverse transcription reaction (Promega, USA). The specific primer of BVDV, as described previously, was used to conduct the real-time PCR (20). In brief, a 135-bp fragment from the 5′UTR of BVDV was amplified: 5′UTR forward, 5′-GGTAGCAACAGTGGTGAGTTC-3′, and 5′UTR reverse, 5′- CTCAGGTTAAGATGTGCTGTG-3′. The qPCR reactions were performed with the GoTaq qPCR master mix (Promega, USA) in the Bio-Rad iQ5 Real-Time PCR System (Bio-Rad Laboratories, USA). A 91-bp fragment of bovine β-actin mRNA in each sample was amplified and used as the endogenous control (BBA forward, 5′-CCCACACGGTGCCCATCTAT-3′, and BBA reverse, 5′-CCACGCTCCGTGAGGATCTTC-3′), in order to normalize the target amplification data (21). The 2–ΔΔCT method was used to perform the relative quantification of RNA of treated cells compared with that of untreated and infected cells. Each sample was tested in triplicate, including the positive and negative controls.

### Western Blot Analysis

Treatment cells were washed using the icy PBS, and total protein samples were extracted using the RIPA Lysis and Extraction Buffer (Thermo Fisher Scientific, USA). According to the instructions of the manufacturer, after being centrifuged at 13,000 rpm for 20 min, concentration of proteins in supernatants was detected using a BCA Protein Assay Kit. Proteins were equally separated on 12–15% SDA-PAGE and then moved onto the PVDF membrane (Merck Millipore, USA). After being blocked in 5% skimmed milk for 2 h, the membrane was incubated with the anti-BVDV E2 antibodies (VMRD, USA) at 4°C overnight and washed, and then incubated with the HRP-conjugated goat anti-mouse IgG (Sigma, USA) at room temperature for 1 h. Signals were developed with an ECL Detection Kit (Thermo Fisher Scientific, USA).

### Rb2 and Rb3 Effect on Viral Replication

To expound the stage of viral replication at which ginsenoside Rb2 and Rb3 exerts their activity, three different assays were performed as described previously (22, 23) with some modifications. MDBK cells were treated with Rb2 and Rb3 before virus infection (pre-treatment group), after infection (post-treatment group), or virucidal group (pre-treatment and post-treatment group) (**Figure 6A**). Rb2 and Rb3 were always used at the non-cytotoxic concentration (200 μg/mL) and the cpBVDV strain C24V titration was always used at 0.05 MOI.

The virucidal assay was conducted with equal amounts of BVDV and ginsenoside. The mixtures were incubated in the microcentrifuge tubes at 37°C for 2 h, and then they were added into the MDBK cell monolayers. The pre-treatment and post-treatment groups were conducted in the procedures that MDBK cell monolayers were incubated with ginsenoside or BVDV virus at 37°C for 2 h. After removal of the liquid, the BVDV or ginsenoside was added. Virus and DMSO were also included on each plate as controls. The plates were incubated at 37°C for 72 h, followed by fixation and staining with BVDV specific antibody labeled by FITC (VMRD, USA).

### Time of Addition Effect of Rb2: Effect on Viral RNA Synthesis

MDBK cells were seeded in 12-well plates and cultured overnight, then infected with cpBVDV strain C24V at a MOI of 0.05, then Rb2 (200 μg/mL) was added at different time points after infection and the cells were further incubated to complete 24 h of infection. All samples were processed for viral RNA analysis.

### Rb2 Effect on CSFV

Using the FA-based virus inhibition assay, the antiviral efficacy of Rb2 against CSFV was investigated.

CSFV Shimen strain with 0.05 MOI was separated and added into the ST cell monolayers and allowed to inoculation for 2 h at 37°C. After inoculation, cells were washed with PBS and serial dilutions of ginsenoside Rb2 was added. Concentrations of compounds were from 25 to 200 μg/mL. Uninfected cell and virus treated with DMSO were used as controls. The plates were incubated at the temperature of 37°C for 72 h, cells were fixed with icy stationary liquid (acetone:methanol = 3:1) at 4°C for 20 min. The cells were further incubated with mouse anti-CSFV E2 mAb WH303 at 37°C for 1 h, washed three times with PBS and incubated with FITC-conjugated rabbit anti-mouse IgG (Sigma, USA) at 37°C for 30 min. After three washes, immunofluorescence was observed using a fluorescence microscope.

### Luciferase Reporter Constructs

The BVDV and CSFV IRES were cloned into the firefly luciferase plasmids pGL4.20 (Promega, USA), respectively. The specific primer for CSFV was described previously (24). The BVDV primer sequences were: F: 5′CCGAGCTCGTATA CGAGAATTT GCCTAGGAC-3′; R: 5′ CCC AAGC TTGGCATAAACAGGTTCTTCCACCC3′. Similar 470 bp fragments of the BVDV were amplified and subcloned into the pGL4.20 using restriction enzymes with *Hind*III and *Sac*I. All reconstructed plasmids were sequenced at Invitrogen Co., Ltd.

### DNA Transfection

The 293T cells were grown in 96-well plates until 80% confluence, reconstructed plasmids and Renilla luciferases plasmids pGL4.74 (Promega, USA) were co-transfected with the X-treme GENETM HP DNA transfection reagent (Roche, Philadelphia, PA), using standard operation, respectively. The parental vector pGL4.20 and pGL4.74 co-transfected group served as mock controls. The cells were allowed to inoculate for 4 h at 37°C. After inoculation, cells were washed with PBS, added with the ginsenoside Rb2 (200 μg/mL) and incubated at 37°C for 48 h. The luciferase activity was determined using the dual luciferase reporter assay system (Promega, USA).

### Statistical Analysis

Student's *t*-test was conducted to examine the statistical differences among ginsenoside treatment groups, virus control, DMSO control, and mock groups. Expression of results was in the form of mean ± standard deviation, representing three independent experiments. Statistical analyses were conducted with GraphPad Prism 6 (GraphPad Software, San Diego, CA). In the case of a value of p < 0.05, it was regarded statistically significant.

## Results

### Cytotoxicity of Compounds

**Figure 1:**
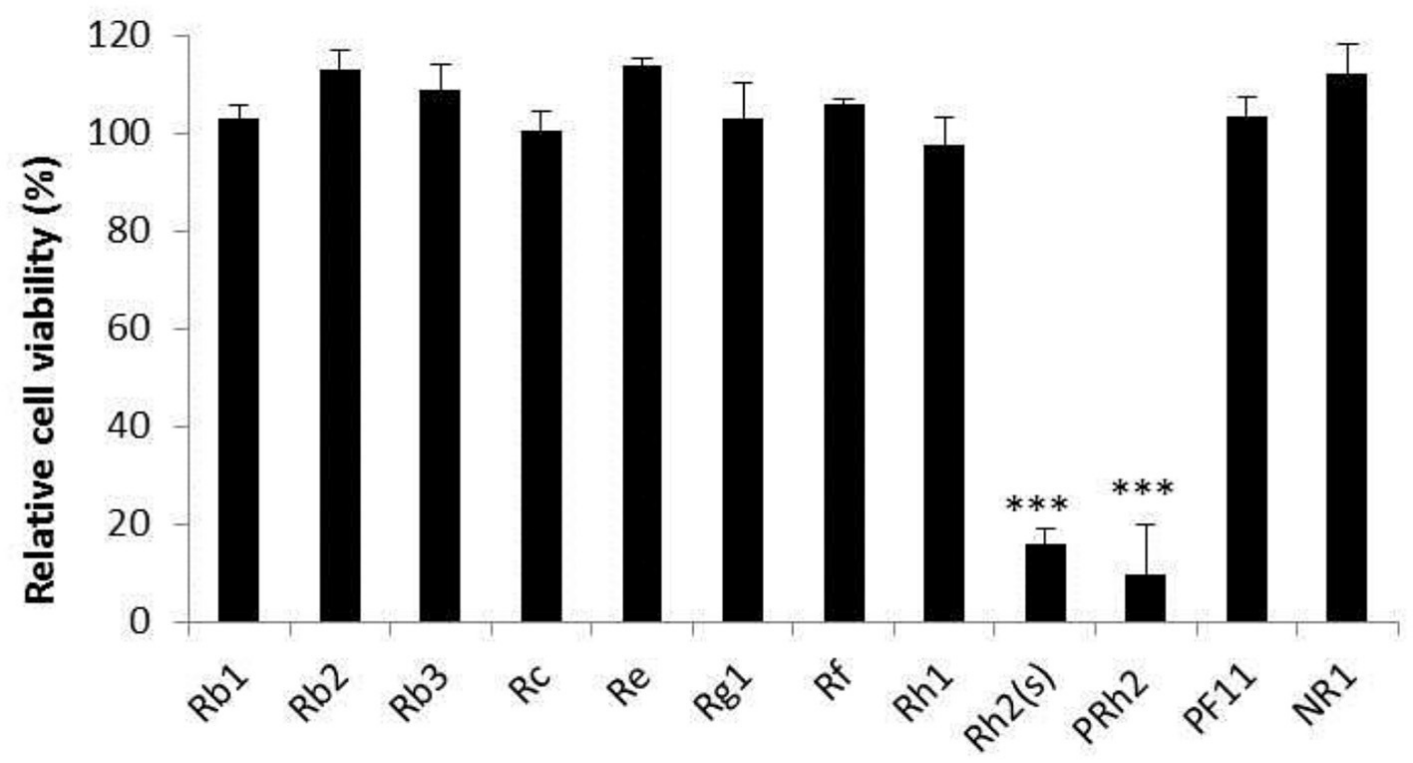
Cytotoxicity of compounds. The result of cell viability is expressed as the percentage of control. The data are expressed as the mean of three experiments. ***p < 0.001. PRh2, pseudoginsenoside Rh2; PF11, pseudoginsenoside F11; NR1, notoginsenosideR1 (NR1).

Most compounds had no effect on the cell viability at 200 μg/mL, while ginsenoside 20(S)-Rh2 and pseudo ginsenoside Rh2 reduced the viability of MDBK cells significantly (Figure 1). Therefore, ginsenoside 20(S)-Rh2 and pseudo ginsenoside Rh2 were not included in further experiments.

### Cytopathic Effect Inhibition by the Compounds

**Figure 2:**
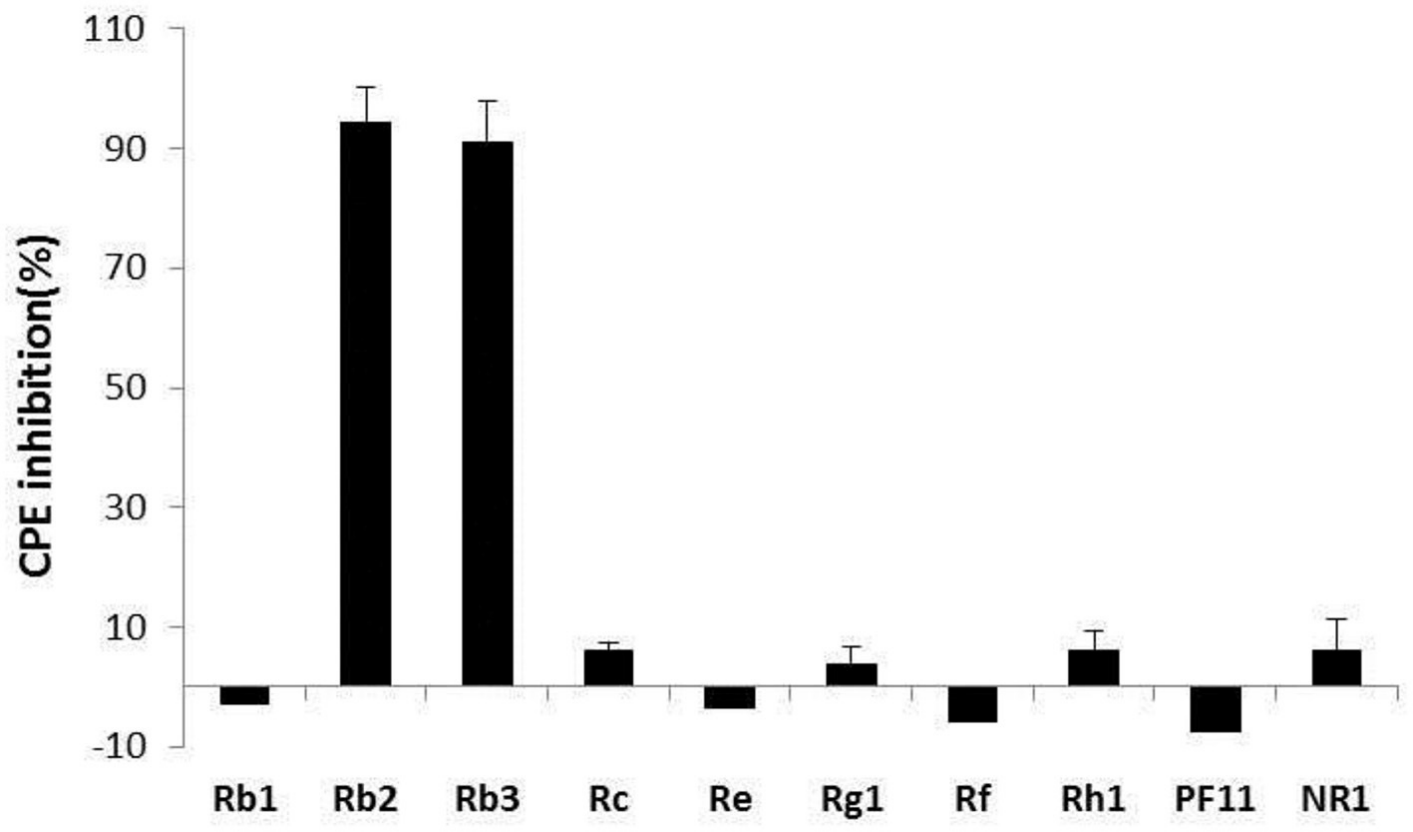
Antiviral activity of compounds. All compound concentrations were used with 200 μg/ml, and uninfected and virus infected cells were used as control. The data are expressed as the mean of three experiments.

**Figure 3:**
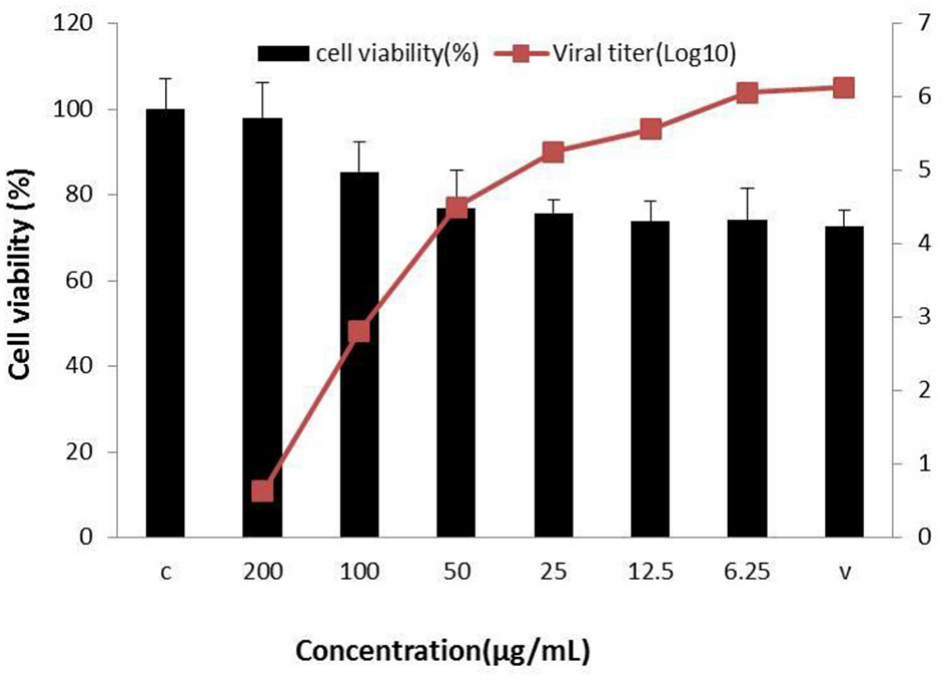
Ginsenoside Rb2 antiviral activity against bovine viral diarrhea virus (BVDV). All serial dilutions Rb2 concentrations were used and uninfected and virus infected cells were controls.

**Figure 4:**
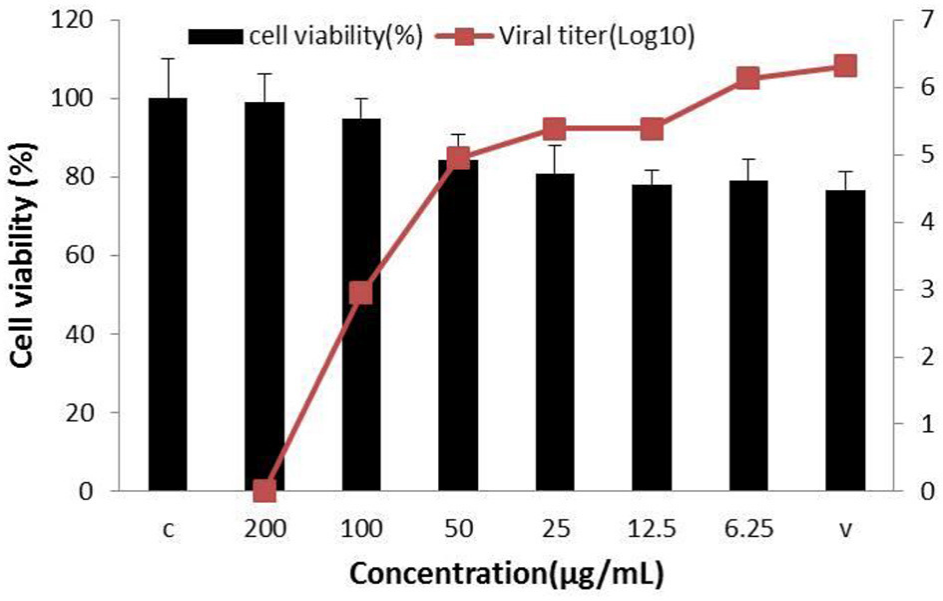
Ginsenoside Rb3 antiviral activity against bovine viral diarrhea virus (BVDV). All serial dilutions Rb3 concentrations were used and uninfected and virus infected cells were controls.

Concerning the antiviral tests undertaken on the 10 compounds, ginsenoside Rb2 and Rb3 significantly inhibited BVDV induced CPE. CPE inhibition was 94.5 and 91.2% at the 200 μg/mL concentration. All other compounds demonstrated a limited or no inhibitory effect on CPE (Figure 2). To further determine the antiviral effect of ginsenoside Rb2 and Rb3, and calculate the EC_50_, different concentrations of Rb2 and Rb3 (6.25–200 μg/mL) were added into the BVDV infected MDBK cells. The viral titer and CPE inhibition were inversely proportional to the ginsenoside Rb2 and Rb3 concentrations (Figures 3, 4). The EC_50_ of Rb2 was 57.76 μg/ mL, that of Rb3 was 60.25 μg/ mL (Table 1).

**Table 1:** The structure and antiviral activity of Rb2 and Rb3.

**Compound**	**Structure**	**MDBK CC_**50**_ (μg/mL)**	**BVDV EC_**50**_ (μg/mL)**	**SI(CC_**50**_**/EC**_**50**_)**
Rb2	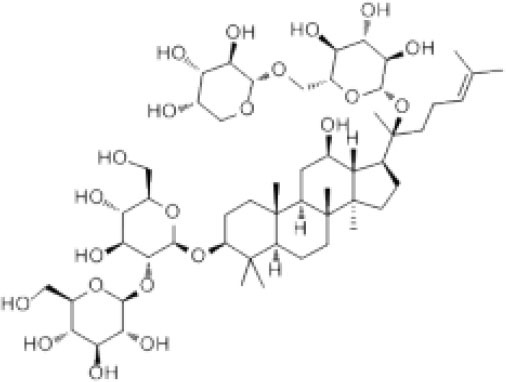	>200	57.76	>3.46
Rb3	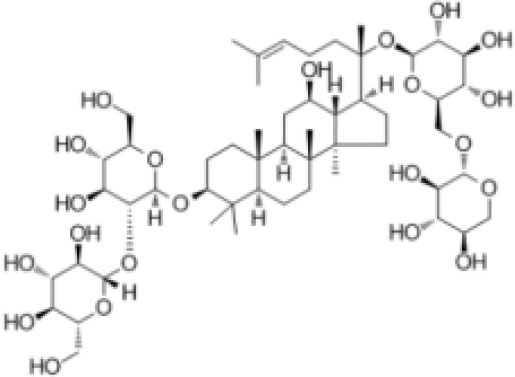	>200	60.25	>3.32

### Antiviral Activity of Ginsenoside Rb2 and Rb3 Against BVDV

**Figure 5:**
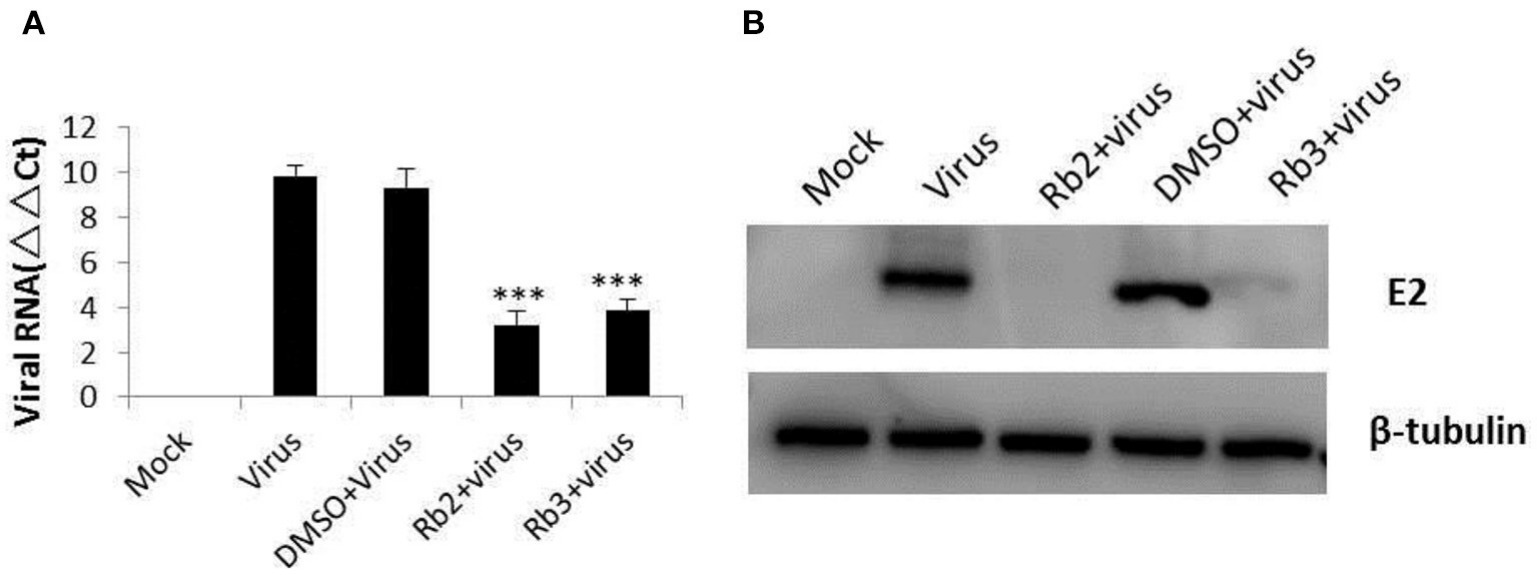
Effect of ginsenoside Rb2 and Rb3 on BVDV replication. **(A)** Real-time PCR analysis of the viral RNA. **(B)** Western blot analysis of BVDV E2 protein expression. Uninfected cells and DMSO-treated cells were used as control. ***p < 0.001.

BVDV replication was significantly inhibited by Rb2 or Rb3, as shown by reduction of viral RNA levels when compared to DMSO (Figure 5A). Similar results were obtained on BVDV E2 protein with Western blot (Figure 5B). Compared with the virus control and DMSO treated group, there is almost nothing or slight bands in the Rb2 and Rb3 treated groups.

### Rb2 and Rb3 Effect on Viral Replication

**Figure 6:**
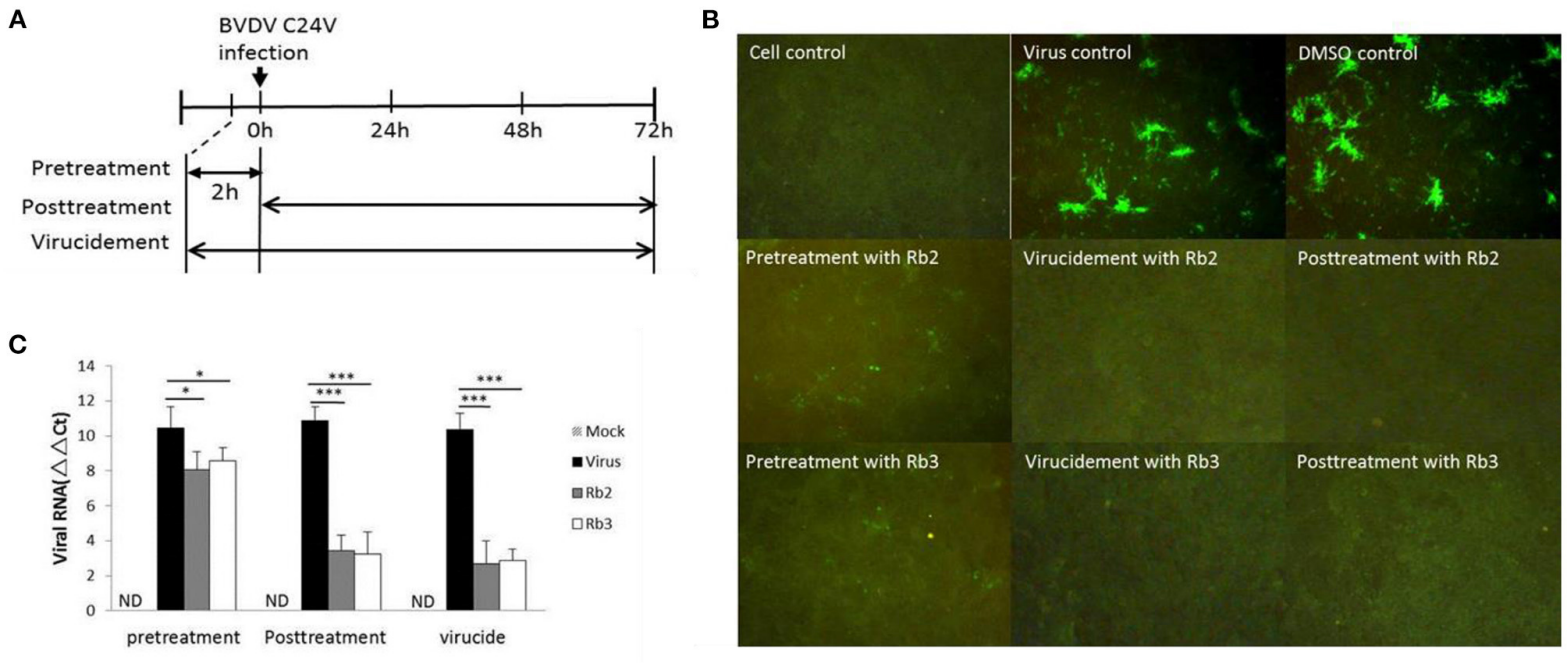
Stage of antiviral activity against cpBVDV C24V by ginsenoside Rb2 and Rb3. **(A)** A schematic diagram describing the experimental scheme for measuring the stage at which ginsenoside exerts its effects. **(B)** The FA result of plaque reduction assays. **(C)** Changes in viral RNA level. *p < 0.05; ***p < 0.001.

The different assays performed to elucidate the mechanism of action of the ginsenoside Rb2 and Rb3, applied at different periods, showed that Rb2 and Rb3 completely suppressed BVDV infection with virucidal assay and post-treatment of cells. Pre-treatment of cells assay also showed effects on BVDV infection, but the inhibitory effect was weaker than other assays (Figures 6B,C).

### Time of Addition Effect of Rb2: Effect on Viral RNA Synthesis

**Figure 7:**
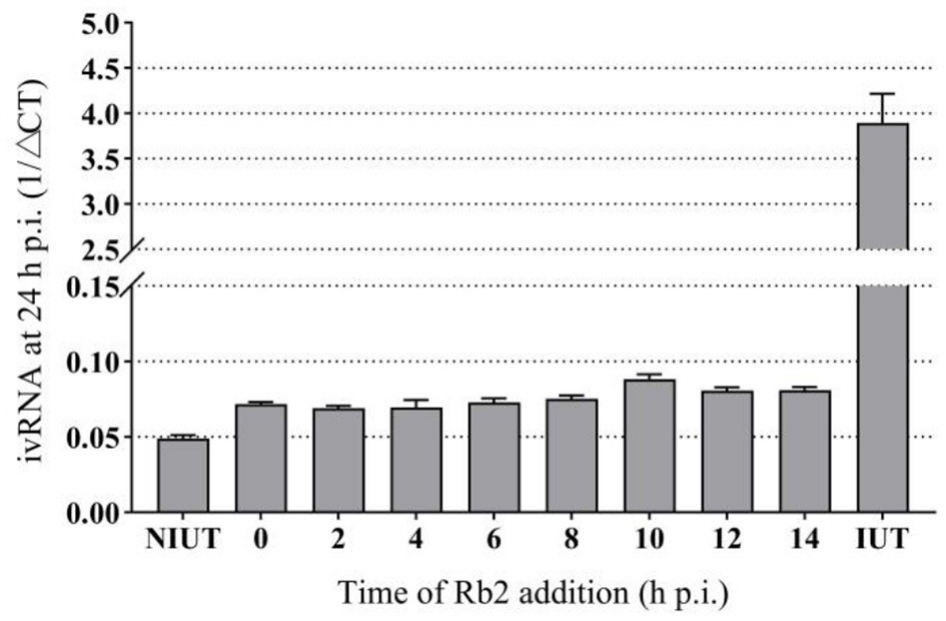
Effect of Rb2 addition on viral RNA synthesis.

In order to give a better perspective about the time of action of Rb2, time of drug addition experiments were carried out. A concentration of Rb2 of 200 μg/mL was added at different points of time after infection, and then viral replication was evaluated as the yield of viral RNA synthesis. Viral RNA was markedly reduced when Rb2 was added during the first 8 h, after which a gradual reduction in drug inhibition was observed. The antiviral effect of Rb2 can be exerted within in 14 h p.i. (Figure 7).

### Rb2 Effect on CSFV

**Figure 8:**
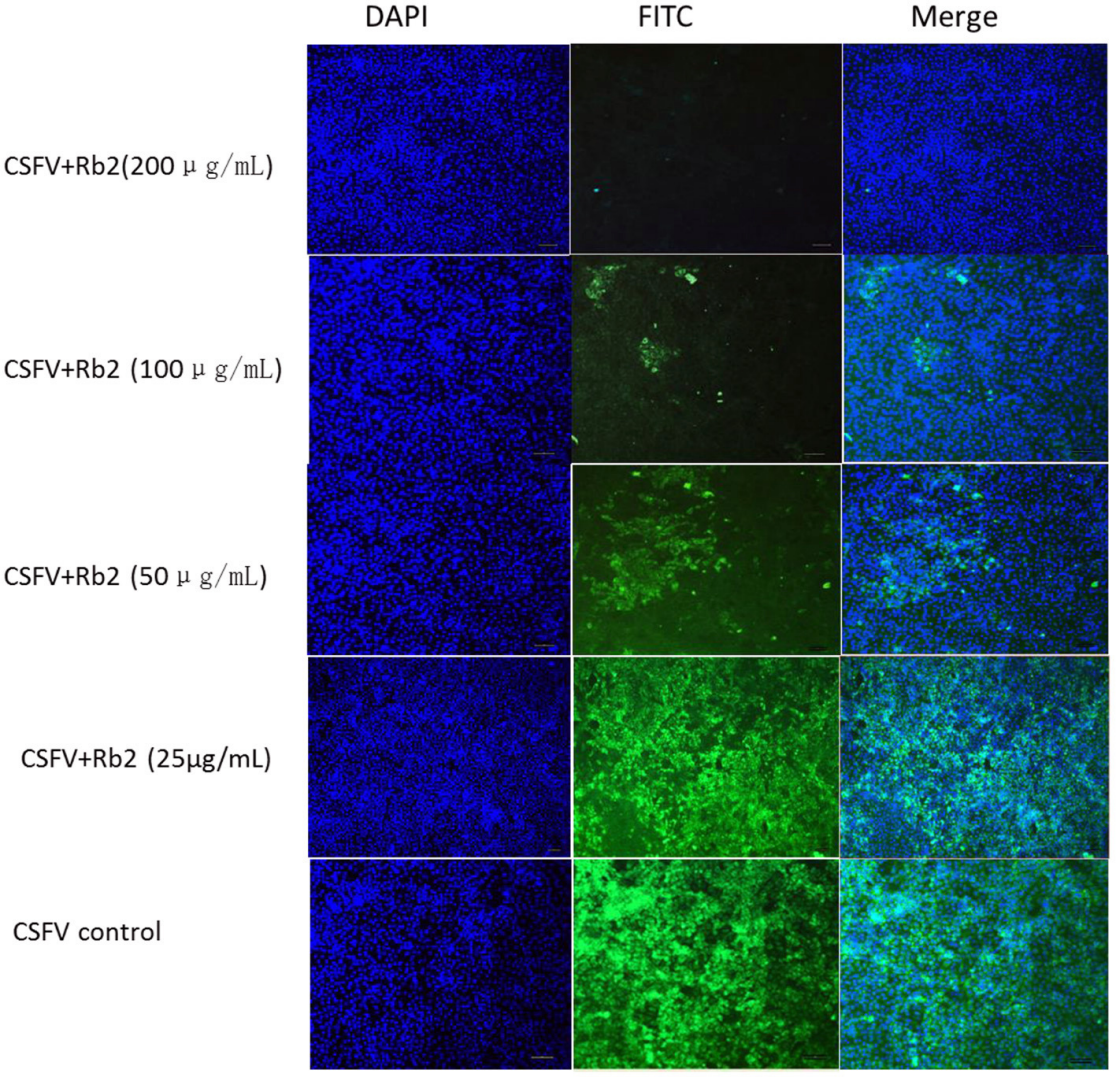
The effect of Rb2 on CSFV Shimen with IFA.

To further understand the ginsenoside Rb2 antiviral activity for other pestiviruses, CSFV Shimen was investigated. Using the FA-based virus inhibition assay, ginsenoside Rb2 demonstrated to have a similar inhibition ability on CSFV to that observed on the BVDV reference strain CV24V (Figure 8).

### Effect of Ginsenoside Rb2 on the *Pestivirus* IRES

**Figure 9:**
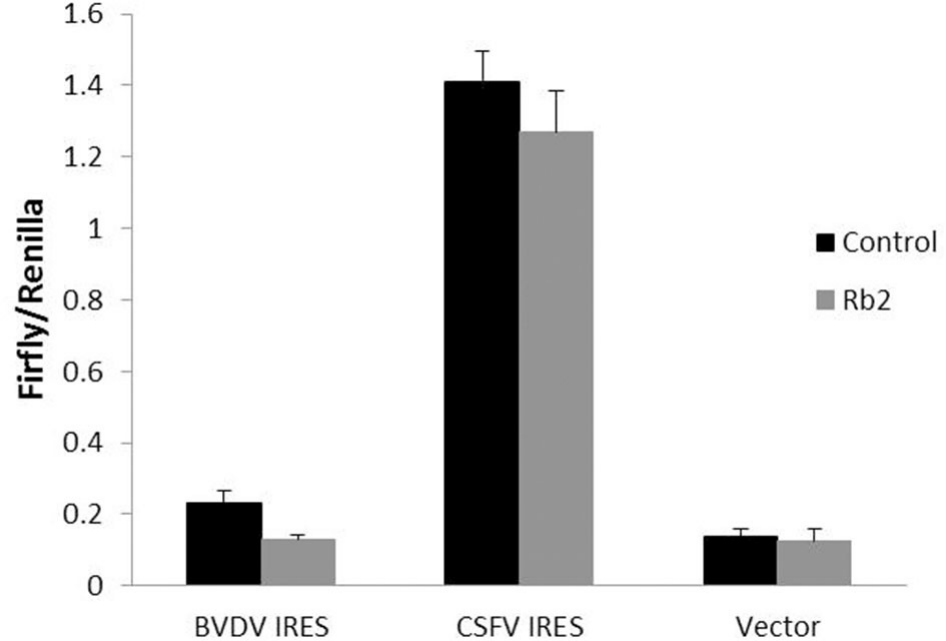
The effect of Rb2 on the activity of IRES of BVDV and CSFV with dual-Luciferase assay. **p < 0.01.

Direct antiviral activity of ginsenoside Rb2 at the level of *Pestivirus* IRES mediated translation in 293T cell culture was investigated. The translation efficiency in 293T cells was analyzed by a reporter RNA encoding Flufly under the *Pestivirus* IRES control, in the presence or absence of the Rb2. As shown in Figure 9, the luciferase activity (Flufly/Renilla) in the presence of Rb2 was significantly reduced than that of the control in absence of Rb2. But the luciferase activity of vector was not changed in the presence or absence of Rb2. These results suggest that ginsenoside Rb2 can inhibit the translation process mediated by the *Pestivirus* IRES.

## Discussion

Ginseng (Panax ginseng) is often used as herbal tonic to increase immunity and reduce stress. These effects are attributed to ginsenosides, major pharmacological components. Although the antiviral activities of ginsenosides have also been reported in several previous studies, the mechanisms underlying the antiviral activity have not yet been elucidated. Several studies about the antiviral mechanism of ginsenosides focused on the immunomodulatory action. The Kang Laboratory (Georgia State University, GA) has published several studies on the immunomodulatory and antiviral effects of KRG extract (RGE) on RSV. Their results indicated that RGE possesses an immunomodulatory effect by balancing Th1 and Th2 immune responses, and protects the host from severe pulmonary inflammation upon FI-RSV immunization and RSV infection (25). The Kang et al. report showed that ginsenoside Rb1 is an immune-stimulatory agent with antiviral activity against enterovirus71. They demonstrated the effective antiviral activities of Rb1 against EV71 *in vitro* and *in vivo*. Furthermore, Rb1 treatment could induce high cellular and humoral immune responses *in vivo*. Meanwhile, Rb1 contributed to the enhanced type I IFN responses and IFN-β knockdown reversed the antiviral activity of Rb1 *in vitro* (26).

The antiviral mechanism of ginsenoside was scarcely studied, and focused on the quantification of inhibitory effect on virus growth, thus indirectly estimating the inhibition of the virus translation process. To investigate if ginsenosides have the same inhibitory effect on virus translation as other synthetic antiviral drugs, we studied the antiviral effect of Rb2 on *Pestivirus* IRES. It is known that the 5′ UTR of *Pestivirus* and HCV RNA genome used for IRES mediated translation, many drugs and antibodies were designed and synthesized according to it (27, 28). The effect of Rb2 on the *Pestivirus* IRES was evaluated through luciferase reporter assay. In the presence of Rb2, the luciferase activity was significantly reduced than that of the control in the absence of Rb2. Compared to CSFV IRES, the luciferase activity in BVDV IRES was lower because the luciferase reporter plasmid of BVDV IRES deleted the 5′ boundary, reducing the IRES function (29). But it did not affect the inhibitory effect of Ginsenoside Rb2. These results suggest that ginsenoside Rb2 could inhibit the translation process mediated by the *Pestivirus* IRES. However, further research is needed to be carried out.

The interest of the observations reported in this study is not necessarily directly related to the control or the prevention of the diseases caused in animals by the pathogens used as model (CSFV and BVDV). While for pestiviruses, prophylactic means are available, no curative compounds can be applied to infected animals. Nevertheless, the current strategy is focused on the eradication of these pathogens to progressively gain an officially free status. In addition, for BVDV, measures rely on the identification and elimination of persistently infected immunotolerant animals, and for CSFV, when the disease is eradicated in a country or zone, stamping out policy is strictly applied.

Bovine-derived biological materials are important to medicine and other biological applications. But contamination is frequently reported with BVD-associated viruses. Contamination of FBS leads to contamination of biological products that use FBS for their production, including cell cultures and vaccines. BVDV contamination can affect the outcome of cell-culture-based research and diagnostic procedures, resulting in misinterpretation of research data or an incorrect diagnosis. Vaccine contamination may not only influence the results of vaccination but may also lead to new infections, causing serious BVD outbreaks (30). With concern to biological product contamination by BVDV, antiviral properties of ginsenosides might be taken into account with a possible use in the framework of laboratory testing and biological products manufacturing activities to counter occurrence of adventitious pestiviruses. However, the problem will be resolved through the application of stringent laboratory safety practices and the compulsory use of biologicals such as bovine fetal serum derived only from officially BVDV-free animals, guaranteed by relevant health competent authorities, and not based on claims from manufacturers. Official free status is contemplated by the recent EU regulations 429/2016 and 689/2020. Possibly, in veterinary medicine, the use of antivirals for pestiviruses might be restricted to highly valuable animals, in order to reduce the impact of the infection on their health and welfare.

HCV is the main causative agent of chronic hepatitis in humans, responsible for more than 185 million infections worldwide. Not only are vaccines absent, but the treatment of hepatitis C has improved only over the past decade. Initially, with interferons and ribavirin, cure rates did not exceed 60% (31, 32), and almost all of them had side effects so severe that some people abandoned the treatment. Highly effective, well-tolerated therapies are now available. However, the therapeutic constraint is represented by very high costs (up to about 97,000 USD for a 12-week treatment). BVDV is genetically similar to HCV in structure and, in general, both of them can produce chronic infections in their hosts, respectively. Due to the limitations in the discovery and development of HCV drugs, compounds with the antiviral activity against BVDV are regarded as a surrogate model system for efforts to discover drugs effective against HCV (33). Therefore, the search for antiviral properties of ginseng using animal models may offer alternatives for the cure and possibly also the prevention of the disease in humans.

In this study, we demonstrated that ginsenoside Rb2 and Rb3 have antiviral effects on inhibition BVDV replicated in MDBK cell. Furthermore, ginsenoside Rb2 and Rb3 showed antiviral potency, thus, representing potentially useful antiviral compounds for controlling contamination in biological products.

## Data Availability Statement

The original contributions presented in the study are included in the article/supplementary material, further inquiries can be directed to the corresponding author/s.

## Author Contributions

BT, NS, YJ, KL, and QW participated in conducting experiments and analysis of biophysical data. MG participated in editing the manuscript. SC and YW participated in the design of the study. SZ proposed the original idea, designed and supervised the experiments, and edited the manuscript. All authors read and approved the final manuscript.

## Funding

This work is funded by National Natural Science Foundation of China (31602093), National Public Welfare of China for Agriculture Special Purpose (CAAS-ASTIP-ISAPS-2021038), and the basic scientific research funding of the Chinese Academy of Agricultural Sciences (0032014016).

## Conflict of Interest

The authors declare that the research was conducted in the absence of any commercial or financial relationships that could be construed as a potential conflict of interest.

## Publisher's Note

All claims expressed in this article are solely those of the authors and do not necessarily represent those of their affiliated organizations, or those of the publisher, the editors and the reviewers. Any product that may be evaluated in this article, or claim that may be made by its manufacturer, is not guaranteed or endorsed by the publisher.

## Abbreviations

BVDV: bovine viral diarrhea virus
CSFV: classical swine fever virus
HCV: hepatitis C virus
ORF: open reading frame
UTR: untranslated regions
IRES: internal ribosome entry site
FBS: fetal bovine serum
PRh2: pseudo ginsenoside Rh2
PF11: pseudo ginsenoside F11
NR1: notoginsenoside R1
DMSO: dimethyl sulfoxide
CPE: cytopathic effect
EC50: 50% effective concentration.
